# Geographically varying relationships of COVID-19 mortality with different factors in India

**DOI:** 10.1038/s41598-021-86987-5

**Published:** 2021-04-12

**Authors:** Asif Iqbal Middya, Sarbani Roy

**Affiliations:** Department of Computer Science and Engineering, Jadavpur University, Kolkata, 700032 India

**Keywords:** Health sciences, Risk factors

## Abstract

COVID-19 is a global crisis where India is going to be one of the most heavily affected countries. The variability in the distribution of COVID-19-related health outcomes might be related to many underlying variables, including demographic, socioeconomic, or environmental pollution related factors. The global and local models can be utilized to explore such relations. In this study, ordinary least square (global) and geographically weighted regression (local) methods are employed to explore the geographical relationships between COVID-19 deaths and different driving factors. It is also investigated whether geographical heterogeneity exists in the relationships. More specifically, in this paper, the geographical pattern of COVID-19 deaths and its relationships with different potential driving factors in India are investigated and analysed. Here, better knowledge and insights into geographical targeting of intervention against the COVID-19 pandemic can be generated by investigating the heterogeneity of spatial relationships. The results show that the local method (geographically weighted regression) generates better performance ($R^{2}=0.97$) with smaller Akaike Information Criterion (AICc $=-66.42$) as compared to the global method (ordinary least square). The GWR method also comes up with lower spatial autocorrelation (Moran’s $I=-0.0395$ and $p < 0.01$) in the residuals. It is found that more than 86% of local $R^{2}$ values are larger than 0.60 and almost 68% of $R^{2}$ values are within the range 0.80–0.97. Moreover, some interesting local variations in the relationships are also found.

## Introduction

The novel coronavirus disease (COVID-19) has spread rapidly to all parts of the world, causing almost 2.5 million deaths as of mid-February 2021^1^. Because of its unpredictable nature and lack of appropriate medications, COVID-19 is now a global health concern. There is unprecedented urgency to investigate the major factors that are related to COVID-19 death. In this context, recent studies are focusing on exploring person-specific risk factors for COVID-19-related health outcomes^2–4^. Also, there are research works that examine the association of COVID-19-related health outcomes with different socio-economic, environmental, and region-specific factors^5–7^. These factors play a very important role in determining the patterns of COVID-19 mortality.

Both global and local models can be utilized to explore the above-mentioned associations. A global model comes up with a geographically constant relationship across the entire geographic space. On the other hand, a local model can capture the local relationships that can vary across the geographic space. Most of the studies that focus on exploring the relationship of COVID-19 cases with different possible risk factors are based on global models (e.g. Ordinary Least Square)^8,9^. But, the global models assume that the associations between the independent variables and the dependent variable are stationary (i.e. homogeneous) throughout the study area. Besides, these models also assume that there is no spatial autocorrelation in the dataset. Eventually, they yield estimates of the parameters that reflect average behaviour^10^. But, in reality, the relationships between the dependent and the independent variables may not be homogeneous and can be geographically varying^11^. Therefore, such models usually suffer from low accuracy especially in those locations where weak association exists between dependent and independent variables. Now, various local techniques can be utilized in order to overcome the above-mentioned shortcomings of the global models. Some widely encountered local spatial statistics include geographically weighted regression (GWR)^12,13^, local Moran’s I^14^, spatial regressions, etc.

As of 24 February 2021, India is the world’s second worst-affected country by COVID-19, with a total number of deaths exceeding 156.7 thousand and a total number of confirmed cases exceeding 11 million^1^. However, in India, no comprehensive study is performed at the local level to investigate geographical relationships between COVID-19 deaths and associated potential factors. To bridge the gap, a local method (GWR) is employed to explore the geographical distribution and associated potential socio-economic, demographic, and environmental factors for COVID-19 deaths. Note that, the GWR model helps us to identify whether there is geographical heterogeneity present in the relationships. Moreover, a comparison between local (OLS) and global (GWR) models are also performed. This paper offers further knowledge and insight into geographical targeting of intervention and control strategies against the COVID-19 epidemic. In summary, the key objectives of this study are (i) to explore the potential socio-economic, demographic, and environmental driving factors for COVID-19 deaths in India; (ii) to investigate geographically varying relationships of COVID-19 deaths with the driving factors by employing local (GWR) model. (iii) comparing the results of the local (GWR) model with the global (OLS) model to validate its suitability.

## Materials and methods

### Data description

**Figure 1:**
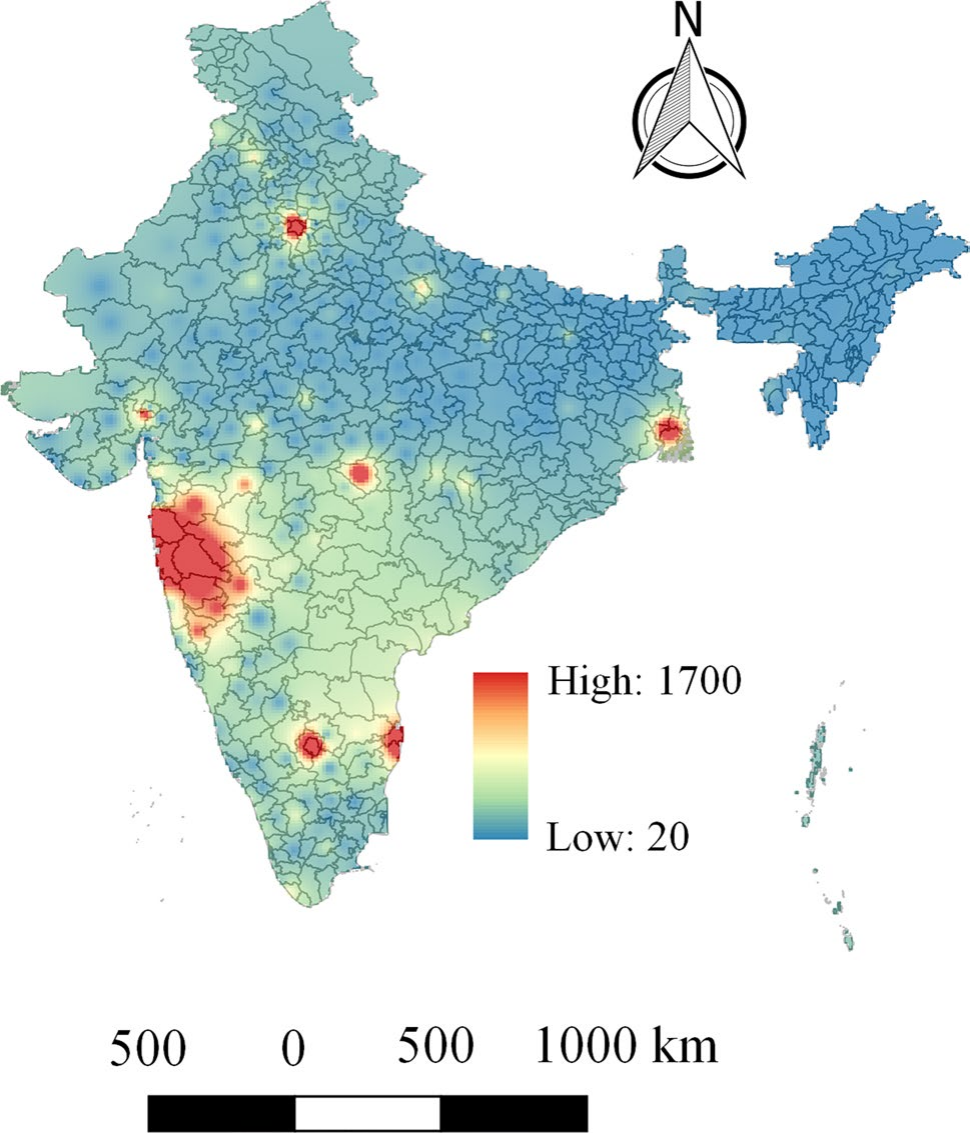
Geographical distribution of COVID-19 deaths across India. The spatially continuous distribution map is generated in QGIS (https://qgis.org/en/site/) by using Inverse Distance Weighting (IDW) interpolation.

The geographical variabilities of COVID-19 deaths are modeled based on the district-level data across India. Note that, the COVID-19 mortality data are acquired for more than 400 districts in India. The geographical distributions of COVID-19 deaths are shown in Fig. 1. The largest number of COVID-19 deaths are observed in the districts of the state Maharashtra. A total of 9 among 28 states contains at least one district that reports more than 1000 COVID-19 deaths. Table 1 summarizes all the raw datasets, their descriptions, the sources including the links from where these data can be found, and potential factors (independent variables) that are extracted from the raw datasets.

**Table 1:** A summary of datasets.

Dataset	Dataset description	Source	Variable name	Variable explanation
COVID-19 data	COVID-19 data from the states and union territories of India up to February 24, 2021.	(i) COVID19INDIA website (https://www.covid19india.org/) (ii) Ministry of Health and Family Welfare, Government of India (https://www.mohfw.gov.in/)	$COVID19\_Death$	District-level COVID-19 death count up to February 24, 2021
Census data	Contains district wise socioecono-mic and demographic data of India	India census, 2011 (https://censusindia.gov.in/)	$Tot\_Population$	District-level total population
			$HH\_Above\_8\_P$	District-level count of total number of households with at least 9 persons
			$Growth\_Rate$	District-level rate at which population increases.
			$Sex\_Ratio$	District-level count of the number of females per 1000 males
			$Age\_Abv\_50$	District-level count of total number of persons with age 50 years or more
			$HH\_With\_TCMC$	District-level count of total number of households having TV, Computer (or laptop), Mobile phone, and Car.
			$Higher\_Edu$	District-level count of total number of persons having higher education
			$P\_Urb\_Pop$	District level percentage of urban population
Environmental air pollution data	Concentration of air pollutants (from January, 2016 to January, 2020) for a total of 130 monitoring stations	(i) Central Pollution Control Board (CPCB), Government of India (https://cpcb.nic.in/)	$PM_{2.5}$	District-level exposure to *PM*2.5, averaged across the period $2016 - 2020$ January, 2016 to January, 2020) for a total of 130 monitoring stations
			$NO_{2}$	District-level exposure to $NO_{2}$, averaged across the period $2016 - 2020$
			$SO_{2}$	District-level exposure to $SO_{2}$, averaged across the period $2016 - 2020$

#### Datasets

Three raw datasets are mainly utilized to investigate geographically varying relationships of COVID-19 deaths with different environmental, demographic, and socio-economic factors. The first dataset includes district wise COVID-19 death counts in India. The cumulative number of COVID-19-related deaths for each district is collected up to February 24, 2021, from the COVID19INDIA website (https://www.covid19india.org/). COVID19INDIA is a crowdsourced initiative to document the COVID-19 data from the states and union territories of India. In this study, the district-level COVID-19 death count is considered as the dependent variable. The second dataset pertaining to environmental pollution includes the daily concentration of different air pollutants (e.g. $PM_{2.5}$, $SO_{2}$, $NO_{2}$, etc.). The concentration of air pollutants (from January 2016 to January 2020) for a total of 130 monitoring stations are obtained from the Central Pollution Control Board (CPCB^15^), INDIA. The third dataset contains socio-economic and demographic data that may have an association with COVID-19 mortality. The district-level socio-economic and demographic data are obtained from the last census in India that was conducted in 2011.

Additionally, the district-level data of each district needs to be linked with the GPS coordinate of the centroid of that district. The dataset containing GPS coordinates of the districts of India are collected from Kaggle (https://www.kaggle.com/sirpunch/indian-census-data-with-geospatial-indexing).

#### Data preparation

From the raw datasets, a total of eleven potential demographic, socioeconomic, and environmental pollution related factors (see Table 1) are selected to explain the district-level geographical variation of COVID-19 mortality. The district-level demographic and socioeconomic factors that are selected in this study are: population; households with at least 9 persons; growth rate; sex ratio; persons with age 50 years or more; households having TV, computer (or laptop), mobile phones and car; number of persons having higher education; the percentage of the urban population. On the other hand, the environmental pollution related variables that are selected are as follows: $PM_{2.5}$ exposure; $NO_{2}$ exposure; $SO_{2}$ exposure.

The district-level long-term exposure to three air pollutants namely *PM*2.5, $NO_{2}$, and $SO_{2}$ are calculated from the raw data of 130 pollution monitoring stations. The mean concentration of each of the above-mentioned air pollutants of all the 130 monitoring stations is computed for the period 2016–2020. For each pollutant, the computed values are spatially aggregated by averaging the values of all monitoring stations of a district. If a district doesn’t contain any monitoring stations, then it’s exposure to that pollutant is computed using Nearest Neighbour interpolation (NNI).

A multicollinearity verification is performed via the Variance Inflation Factor (VIF) to remove unnecessary redundancy among the explanatory variables. VIF can be expressed as follows [Eq. (1)]:

1 $$\begin{aligned} VIF^{k}= \, & {} \frac{1}{1-R_{k}^{2}} \end{aligned}$$

2 $$\begin{aligned} R_{k}^{2}= \, & {} 1- \frac{SSE_k}{SST_k} \end{aligned}$$

where, $R_{k}^{2}$ denotes the coefficient of determination that is computed by regressing the $k^{th}$ variable on remaining explanatory variables. The mathematical expression for $R_{k}^{2}$ is given in Eq. (2). Here, $SSE_k$ and $SST_k$ denote the sum of squares of total variation and sum of squares of errors respectively. Firstly, regression analysis is conducted among all the 11 explanatory variables to compute the VIFs that are shown in Table 2. It is observed that the variable *HH_Abv_8_P * has high Variance Inflation Factor (VIF = 12.4). Now, if VIFs are larger than 10, it indicates that there is multicollinearity^16^. Eventually, the variable *HH_Abv_8_P * is removed from the set of explanatory variables. After that, the regression is again performed on the remaining 10 variables, with the VIFs given in Table 3. Now, it is observed that no VIF exceeds 10 eventually this set of 10 variables can be used for model building.

**Table 2:** VIFs with all the 11 explanatory variables.

Variable	VIF
$Tot\_Population$	7.92
$Growth\_Rate$	1.24
$Sex\_Ratio$	1.30
$HH\_With\_TCMC$	3.91
$HH\_Abv\_8\_P$	12.4
$Higher\_Edu$	3.12
$PM_{2.5}$	2.65
$Age\_Abv\_50$	3.47
$P\_Urb\_Pop$	1.93
$SO_{2}$	1.20
$NO_{2}$	1.52

**Table 3:** VIFs after removing the variable $HH\_Abv\_8\_P$.

Variable	VIF
$Tot\_Population$	3.93
$Growth\_Rate$	1.21
$Sex\_Ratio$	1.32
$HH\_With\_TCMC$	2.96
$Higher\_Edu$	2.94
$PM_{2.5}$	2.35
$Age\_Abv\_50$	2.87
$P\_Urb\_Pop$	1.80
$SO_{2}$	1.19
$NO_{2}$	1.54

### Modeling spatial relationship

In this paper, the OLS (Ordinary Least Square) and GWR (Geographically Weighted Regression) models are utilized to determine the geographical relationship of COVID-19 mortality with potential risk factors.

The OLS method generally attempts to understand the global relationships between the dependent and independent variables. In this case, the regression and its parameters are unchanged over the geographic space. Mathematically, Eq. (3) represents a global regression model as follows:

3 $$\begin{aligned} {\mathscr {Y}}_{i}= \eta _{0} + \sum _{k=1}^{n} \eta _{k} {\mathscr {X}}_{ik} + \delta _{i} \end{aligned}$$

where, ${\mathscr {Y}}_{i}$ denotes the dependent or response variable; ${\mathscr {X}}_{ik}$ is the *i*th observation of *k*th independent variable; $\eta _{k}$ the global regression coefficient for *k*th independent variable; $\eta _{0}$ represent the intercept parameter; and $\delta _{0}$ denotes the error term.

GWR technique extends the global regression [Eq. (3)] by enabling local parameter estimation^13^. It allows regression coefficients to be a function of geographical location. In other words, the regression coefficients are quantified independently in different geographical locations. A GWR model [Eq. (4)] can be represented as follows:

4 $$\begin{aligned} {\mathscr {Y}}_{i} = \xi _{i0} + \sum _{k=1}^{n} \xi _{k} (\mu _{i},\nu _{i}) {\mathscr {X}}_{ki} + \delta _{i} \end{aligned}$$

where, ${\mathscr {Y}}_{i}$, ${\mathscr {X}}_{ki}$, and $\delta _{i}$ denote the dependent (or response) variable, *k*th independent (or predictor) variable, and error at location *i* respectively; $(\mu _{i},\nu _{i})$ denotes coordinates of location *i*; $\xi _{k} (\mu _{i},\nu _{i})$ represent local coefficient for *k*th predictor at location *i*. Note that, GWR model allows regression parameters to vary continuously across the geographic space. For each location *i*, a set of regression parameters is estimated. The estimation of parameters can be performed as follows:

5 $$\begin{aligned} {\widehat{\xi }}(\mu , \nu )= ({\mathscr {X}}^{T} {\mathscr {W}} (\mu , \nu ) {\mathscr {X}})^{-1} {\mathscr {X}}^{T} {\mathscr {W}} (\mu , \nu ){\mathscr {Y}} \end{aligned}$$

where, ${\mathscr {X}}$ denotes a matrix containing the values of independent variables and a column of all 1s; ${\mathscr {Y}}$ represents a vector of values of the dependent variable; ${\widehat{\xi }}(\mu , \nu )$ is a vector of local regression parameters; ${\mathscr {W}} (\mu , \nu )$ is a diagonal matrix whose diagonal elements represent the geographical weighting of the observations for regression location. The weights in ${\mathscr {W}} (\mu , \nu )$ assigns greater weights to the observations that are closer to the regression point than the observations that are farther away. In this work, the weights are computed using a Gaussian kernel function which is defined as follows:

6 $$\begin{aligned} {\left\{ \begin{array}{ll} w_{ij}=exp \Big [ - \frac{1}{2} \Big (\frac{D_{i}^{j}}{B} \Big )^{2} \Big ], &{} \text {if} ~D_{i}^{j} \le B\\ w_{ij}=0, &{} \text {otherwise} \end{array}\right. } \end{aligned}$$

where, *B* represents the bandwidth and $D_{i}^{j}$ denotes the distance between the regression point *i* and the location of observation *j*. Note that, the bandwidth can be defined either by a fixed number of closest neighbors (known as adaptive bandwidth) or by a fixed distance (known as fixed bandwidth). Golden Section search^17^ is utilized to find the optimum size of the bandwidth for GWR.

#### Performance metrics

The performance of the models are assessed by three metrics namely $R^{2}$, adjusted $R^{2}$, and AICc. Here, AICc is a corrected version of the Akaike Information Criterion (AIC). AICc can be defined as follows^13^:

7 $$\begin{aligned} AICc=N \, ln(2\pi ) + 2N~ln({\hat{\sigma }}) + N \times \Bigg ( \frac{N+tr(S)}{N-2-tr(S)} \Bigg ) \end{aligned}$$

where, *N* denotes the sample size, *S* is the hat matrix, *tr*(*S*) denotes the trace of *S*, and ${\hat{\sigma }}$ represents the estimated standard deviation of the error term. AICc denotes model’s accuracy and lower AICc indicates better model quality. It is usually used to find the best-fit model. The value of $R^{2}$ represents the ability of a model to explain the variance in the dependent variable and therefore a larger $R^{2}$ signifies the better performance of the model. It is computed from the estimated and the actual values of the dependent variable. Moreover, Moran’s I index is computed to investigate the spatial autocorrelation of the model residuals. Mathematically, it is defined as follows:

8 $$\begin{aligned} I=\frac{N \sum _{i=1}^{N} \sum _{j=1}^{N} w_{ij} (y_{i}-{\bar{y}})(y_{j}-{\bar{y}})}{\Big ( \sum _{i=1}^{N} \sum _{j=1}^{N} w_{ij} \Big ) \sum _{i=1}^{N} (y_{i} - {\bar{y}})^{2} } \end{aligned}$$

where, N denotes total number of observations, $y_{i}$ and $y_{j}$ are variable values at location *i* and *j* respectively,${\bar{y}}$ represents the mean value, and $w_{ij}$ denotes a weight between location *i* and *j*. The value of Moran’s I index can vary between − 1 (perfect dispersion) to + 1 (a perfect positive autocorrelation). Note that, a zero value indicates perfect spatial randomness.

#### Model building

Here, a step-wise GWR model selection using AICc is presented that can be utilized to investigate geographically varying relationships of COVID-19 mortality with different driving factors. The following are the steps to build an appropriate GWR model^18^.

- *Step 1* Suppose there are *n* explanatory variables (in our case $n=10$). For each of the explanatory variables, fit a separate GWR model by regressing that variable against the $COVID19\_Death$ variable. Compute AICc for each of the $n=10$ models. Find the model that generates the lowest AICc and permanently include the corresponding explanatory variable in subsequent model building.
- *Step 2* Subsequently select a variable from the remaining ($n-1$) variables, build a model with the permanently included variables along with the newly selected variable. Find the explanatory variable that produces the lowest AICc and permanently include it in subsequent model building. Set $n=n-1$.
- *Step 3* Repeat Step 2 until it is observed that there is no reduction in AICc.

**Figure 2:**
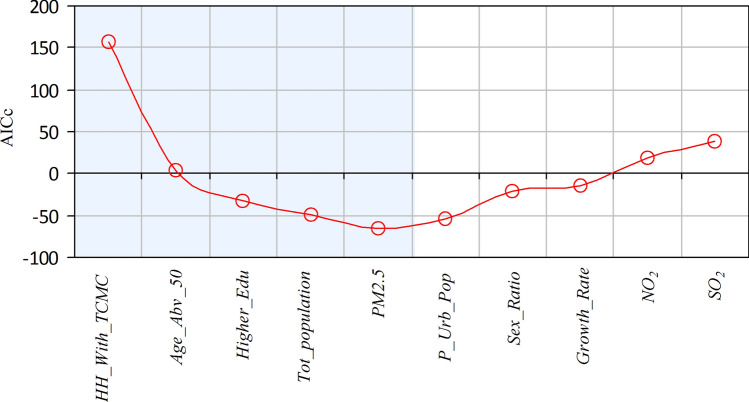
Stepwise variable selection for geographically weighted regression (GWR).

The above-mentioned steps are carried out using MGWR 2.2 software^19^. When calibrating the GWR, an adaptive bisquare spatial kernel is applied. Moreover, in order to select an optimal bandwidth, the Golden Section search^17^ is employed. Figure 2 shows the changes in AICc during the step-by-step selection of explanatory variables for model building. It is observed that after the inclusion of a total of five variables, the AICc values start increasing when further new variables are included. Note that, both a global (OLS) model and a local (GWR) model are calibrated with these five explanatory variables.

## Results

In this section, firstly the performance of the global model (OLS) and local model (GWR) are discussed. Next, the geographically varying relationships of COVID-19 mortality with different factors are presented.

### Performance of OLS and GWR model

A detailed summary of the OLS model is presented in Table 4. The variables *Tot_population*, *HH_With_TCMC*, *Age_ABV_50*, and $PM_{2.5}$ returns significant t values of 2.91, 12.114, − 1.225 and − 2.485 respectively. Moreover, the Moran’s I of the residuals of the global OLS model are also analysed. It is found that there is significant spatial autocorrelation (Moran’s I = 0.348 and p < 0.01). The assumptions of OLS estimation are violated as there exist dependent residuals. Eventually, the GWR model is utilized to show the geographical variations of the relationships with different factors. A detailed summary of the GWR model for the local parameter estimates is presented in Table 5.

**Table 4:** Summary of the global model (OLS) for various socioeconomic, demographic, and environmental pollution related factors.

Variable	Coef. Est	Est Err	t statistic	p-value
Intercept	0.000	0.026	0.000	1.000
$Tot\_population$	0.322	0.11	2.91	0.005*
$HH\_With\_TCMC$	0.675	0.045	12.114	0.000*
$Age\_Abv\_50$	0.125	0.102	– 1.225	0.050*
$Higher\_Edu$	0.136	0.073	1.851	0.064
$PM_{2.5}$	– 0.079	0.032	– 2.485	0.013*

*Significant at 0.05.

**Table 5:** Summary of the local model (GWR) for various socioeconomic, demographic, and environmental pollution related factors.

Variable	Mean	STD	Min	Median	Max
Intercept	0.413	0.292	– 0.789	0.043	1.134
$Tot\_population$	0.152	0.604	– 2.525	0.126	1.385
$HH\_With\_TCMC$	0.520	0.382	– 0.258	0.408	2.550
$Age\_Abv\_50$	0.317	0.648	– 1.016	0.189	2.861
$Higher\_Edu$	– 0.038	0.381	– 1.566	– 0.025	1.488
$PM_{2.5}$	0.101	0.352	– 1.721	0.015	0.842

**Figure 3:**
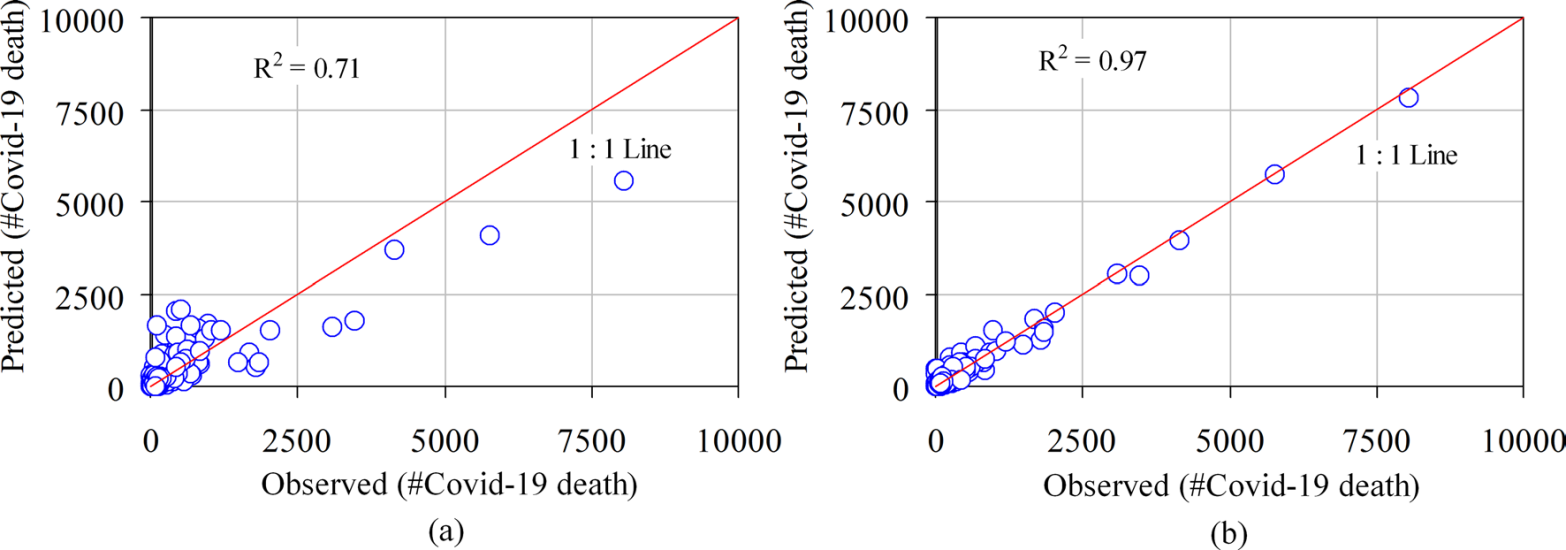
Scatter plot of the observed and the predicted COVID-19 death count using (**a**) global OLS model and (**b**) GWR model.

The performance of OLS and GWR model in terms of $R^{2}$, $Adj \, R^{2}$, and AICc are also provided in Table 6. Moreover, Fig. 3a and b show the scatter plots between predicted and observed COVID-19 death count using the global OLS and GWR models. These figures indicate that the GWR model resulted in a better fit as compared to the global OLS model. This is because, in the case of GWR, the predicted values are closely distributed along the 1:1 line relative to the observed values. The global model explains only 71.9% of the variance of district-level COVID-19 deaths which is increased to 97% if the model is calibrated as GWR by taking into account the local impact of the explanatory variables. Comparing the models in terms of AICc, show that the model fit is greatly enhanced by reducing the value of AICc from 655.835 (OLS model) to -66.42 (GWR model). Moreover, the verification of Moran’s I of the residuals of the GWR model indicates that the residuals are randomly distributed (Moran’s I=-0.0395 and p < 0.01). In other words, the residuals don’t have any significant spatial autocorrelation and eventually, it shows the suitability of GWR over the global model (OLS).

**Table 6:** Performance comparison OLS and GWR models in terms of three performance metrics: AICc, $R^{2}$, and Adjusted $R^{2}$.

Performance metrics	OLS	GWR
AICc	655.835	– 66.42
$R^{2}$	0.719	0.97
$Adj \, R^{2}$	0.715	0.964

### Geographically varying relationships between COVID-19 deaths and the driving factors

**Figure 4:**
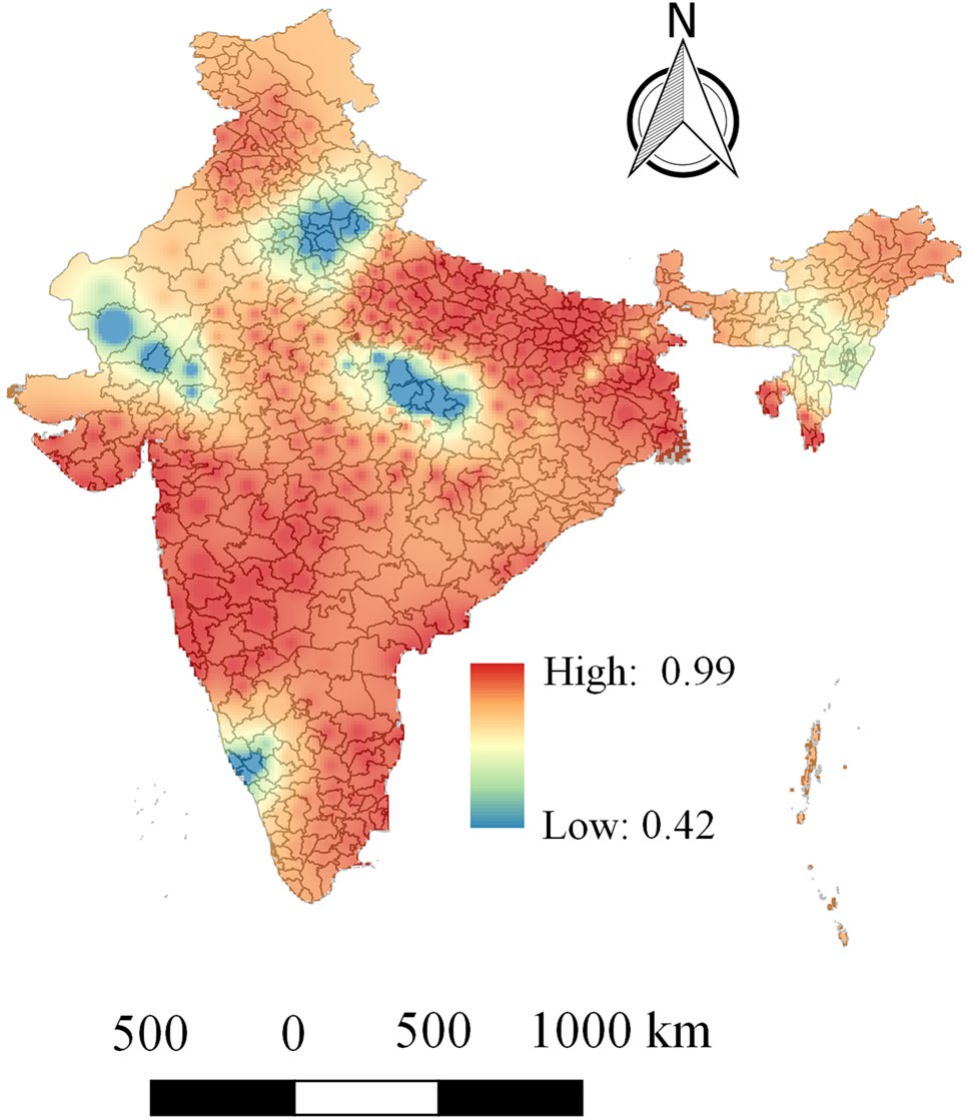
Geographical distribution of $R^{2}$ values for geographically weighted regression (GWR) model.

The geographical distribution of $R^{2}$ is presented in Fig. 4 that shows it varies within a range 0.42–0.97. It is found that more than 86% of local $R^{2}$ values are larger than 0.60 and almost 68% of $R^{2}$ values are within the range 0.80–0.97. Note that, very high $R^{2}$ values are mainly observed in the western and the eastern regions of India. Moreover, low and moderate $R^{2}$ values are mainly distributed over the northern and the southern part of India.

**Figure 5:**
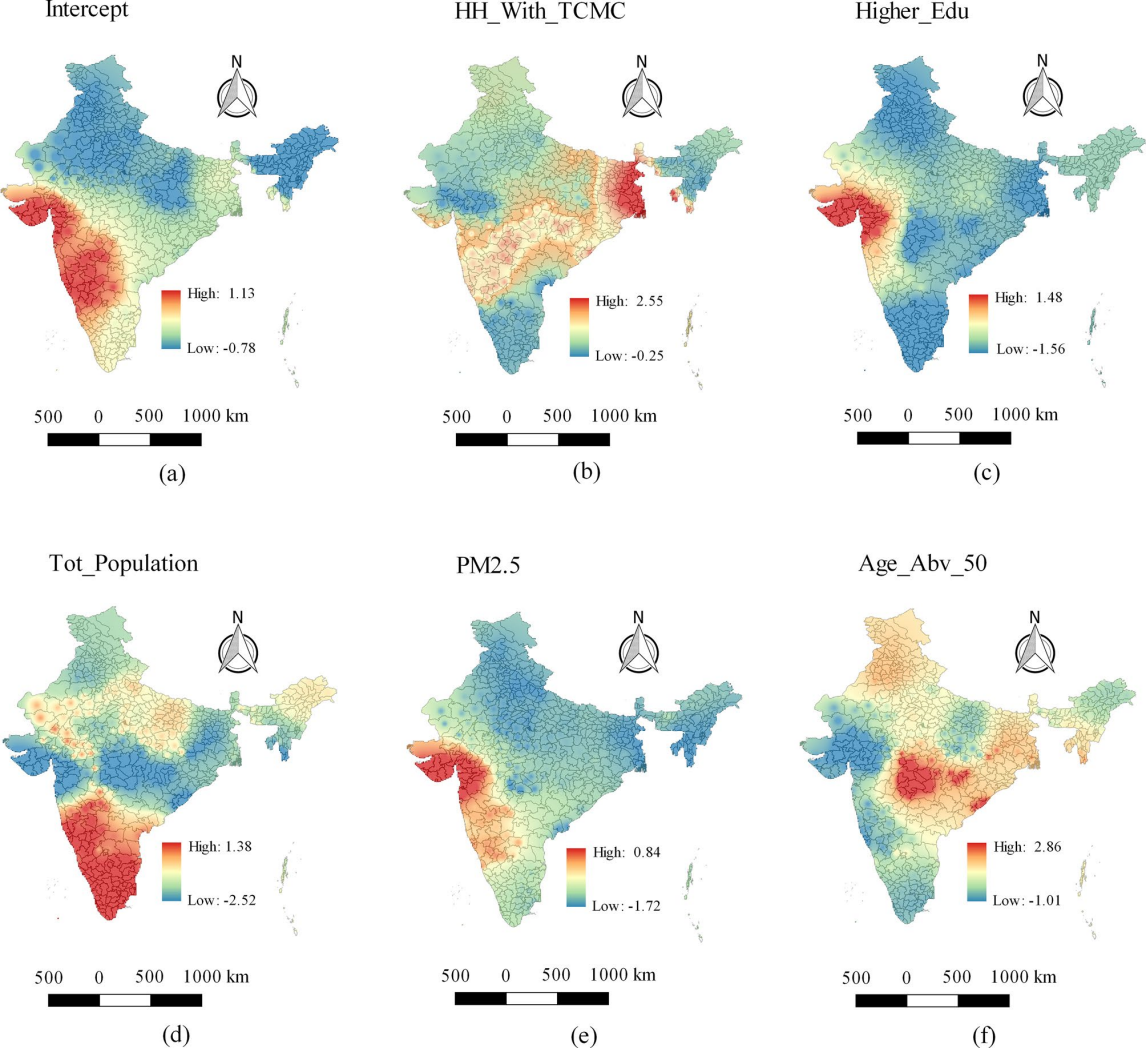
Local parameter estimates of geographically weighted regression (**a**) Intercept (**b**) $HH\_With\_TCMC$ (**c**) $Higher\_Edu$ (**d**) $Tot\_population$ (**e**) *PM*2.5 and (**f**) $Age\_Abv\_50$.

Now, the geographical distribution of local coefficient estimates of the GWR model is provided in Fig. 5 to further reveal the relationship of the explanatory variables with the COVID-19 deaths. It mainly facilitates understanding of the complex relationship that varies over the geographic space. The results of GWR in Fig. 5 not only present positive or negative relationships but also show whether the relationship is strong or weak. A positive relationship indicates that the COVID-19 deaths tend to increase as the value of specific explanatory variable increases. A negative relationship indicates that the COVID-19 deaths tend to decrease as the value of specific explanatory variable increases. Moreover, larger values of a coefficient denote a stronger relationship. In the maps of Fig. 5, the regions having deep red shade denote regions in which the specific variable has a strong positive influence (i.e. strong positive relationship) on COVID-19 deaths.

As shown in Fig. 5a, the GWR model produces local intercept that can vary within the range – 0.78 to 1.13 with a mean of 0.013. In Fig. 5b, the regions with deep red color (mainly the state of West Bengal) denote those areas where the variable $HH\_With\_TCMC$ has a strong positive relationship with COVID-19 death. The variable $Higher\_Edu$ is a strong predictor (See Fig. 5c) for COVID-19 death in some parts of western India (mainly the state of Gujarat), southern India (mainly the states of Tamil Nadu and Kerala), and Eastern India (mainly the state of West Bengal). On the other hand, in the southern and the south-western part of India, a positive relationship between population and COVID-19 death is found (see Fig. 5d). However, in some regions of central and western India (the states of Madhya Pradesh and Gujarat), a strong negative relationship between population and COVID-19 death is also observed. Fig. 5e shows that mainly in the western part of India there is a strong positive relationship between $PM_{2.5}$ and COVID-19 death, whereas in the other parts of India there is no such strong relationship. The explanatory variable $Age\_Abv\_50$ shows a positive relationship in central, eastern, and northern parts of India (see Fig. 5f).

Moreover, Table 7 represents the district-level results of the local model (GWR) for some of the districts that are severely affected by COVID-19 disease. The local $R^{2}$ values revealed district-level variability in GWR model performance. Specifically, the local $R^{2}$ values could be helpful here to see where geographically weighted regression predicts well and where it predicts poorly. It is observed that the GWR model yields high local $R^{2}$ value for most of the heavily affected districts. For instance, very high local $R^{2}$ values are found for the following districts: Pune ($R^{2}=0.982$), Thane ($R^{2}=0.986$), Lucknow ($R^{2}=0.984$), Chittor ($R^{2}= 0.973$), Nasik ($R^{2}=0.986$), Solapur ($R^{2}=0.974$), Kolhapur ($R^{2}=0.972$), Sangli ($R^{2}=0.973$), Satara ($R^{2}=0.978$), Latur ($R^{2}=0.967$), Mumbai ($R^{2}=0.981$), Kolkata ($R^{2}=0.987$), Chennai ($R^{2}=0.978$), Jalgaon ($R^{2}=0.966$), Nanded ($R^{2}=0.960$). On the other hand, moderate local $R^{2}$ values are found for Dharwad ($R^{2}=0.948$), Nagpur ($R^{2}=0.955$), Srikakulam ($R^{2}= 0.920$), Ludhiana ($R^{2}= 0.921$), Guntur ($R^{2}=0.95$), Kurnool ($R^{2}= 0.856$), Coimbatore ($R^{2}=0.934$), West Godavari ($R^{2}= 0.95$), Anantapur ($R^{2}=0.886$), Bhopal ($R^{2}=0.916$), and Krishna ($R^{2}= 0.937$). The lowest $R^{2}$ values are observed for the following districts: Hassan ($R^{2}=0.683$), Indore ($R^{2}=0.798$), and Jaipur ($R^{2}= 0.797$). Note that, for most of the highly COVID-19-affected districts, the variables $PM_{2.5}$ and $HH\_With\_TCMC$ are usually exhibited positive relationships in regression modeling. On the other hand, the variable $Higher\_Edu$ usually exhibits negative relationships for most of the highly affected districts.

**Table 7:** Local $R^{2}$ and district-level parameter estimates by geographically weighted regression for some of the districts of India that are severely affected by COVID-19 disease.

District	Local $R^{2}$	Parameter estimates					
		Intercept	*PM*2.5	$Tot\_population$	$HH\_With\_TCMC$	$Age\_Abv\_50$	$Higher\_Edu$
Pune	0.982	0.219	− 0.221	− 0.311	0.661	− 0.154	0.768
Mumbai	0.981	0.654	0.712	− 0.388	0.859	− 0.074	0.719
Thane	0.986	0.086	0.806	− 0.958	0.716	− 0.084	0.897
Chennai	0.978	0.234	0.421	0.689	1.089	− 0.496	− 0.575
Kolkata	0.987	0.057	− 0.055	− 0.146	1.114	0.658	− 0.152
Nashik	0.986	0.030	0.800	− 0.280	0.742	0.079	0.985
Jalgaon	0.966	0.099	0.357	− 0.389	0.744	0.284	0.853
Nagpur	0.955	0.088	− 0.132	− 1.214	0.685	1.588	− 0.571
Solapur	0.974	0.318	0.476	0.516	0.685	− 0.073	0.241
Kolhapur	0.972	0.238	0.249	0.483	0.791	− 0.142	0.244
Surat	0.976	0.223	0.749	− 1.189	0.481	− 0.127	1.469
Sangli	0.973	0.281	0.399	0.358	0.576	− 0.324	0.127
Ludhiana	0.921	− 0.037	− 0.175	− 0.189	0.287	0.423	− 0.225
Chittoor	0.973	− 0.029	0.102	0.562	0.128	0.117	− 0.286
East Godavari	0.959	0.159	− 0.159	− 0.541	1.078	0.458	− 0.654
Indore	0.798	0.011	0.062	0.141	− 0.180	− 0.120	0.323
Guntur	0.954	0.150	0.116	0.274	− 0.185	0.247	− 0.142
Lucknow	0.984	− 0.043	− 0.104	0.049	0.389	− 0.109	0.284
Satara	0.978	0.264	0.370	0.196	0.793	− 0.222	0.482
Kurnool	0.856	0.229	0.392	0.366	0.371	0.144	0.096
Madurai	0.950	0.143	0.078	0.629	0.162	− 0.059	− 0.651
Anantapur	0.886	0.094	0.167	0.226	0.107	0.258	− 0.125
Dharwad	0.948	0.193	0.283	0.523	0.741	− 0.139	0.105
West Godavari	0.954	0.166	− 0.068	0.029	0.322	0.198	− 0.284
Coimbatore	0.934	− 0.145	0.099	0.578	0.321	− 0.127	− 0.381
Prakasam	0.953	0.137	0.071	0.324	− 0.179	0.314	− 0.101
Bhopal	0.916	− 0.044	0.037	− 0.296	0.475	0.295	0.125
Krishna	0.937	0.151	− 0.074	0.221	0.102	0.194	− 0.214
Latur	0.967	0.348	0.356	0.571	0.598	0.542	− 0.293
Jaipur	0.797	− 0.143	− 0.131	0.019	0.289	0.225	− 0.126
Srikakulam	0.920	0.086	0.108	− 1.146	0.854	1.285	− 0.276
Nanded	0.960	0.255	0.117	− 0.076	0.725	1.108	− 0.508
Dhule	0.966	0.074	0.533	− 0.746	0.385	− 0.278	1.175
Hassan	0.683	0.065	0.214	0.417	0.401	0.112	− 0.419

## Discussion

In order to better understand how different driving factors influence the overall fatalities caused by COVID-19, the geographical distribution of COVID-19-related deaths are investigated. The highest number of COVID-19-related deaths are found primarily in the western part of India (Pune, Thane, Mumbai, Nagpur, Nashik, Raigad, Jalgaon, Kolhapur, Sangli, Satara, Solapur, Ahmedabad, Surat). On the other hand, the number of COVID-19-related deaths is relatively low in the northern and eastern parts of India. This study identified considerable geographical variability of COVID-19 deaths and their heterogeneous relationship at the local level with the driving factors in India. More specifically, the utilization of the GWR method successfully found the geographically varying relationship of COVID-19 mortality with various potential socio-economic, demographic, and environmental pollution related factors. This study reveals five important local factors are significantly related with district-level COVID-19 deaths as follows: (i) population (ii) $PM_{2.5}$ level (iii) households having TV, computer (or laptop), mobile phones and car (iv) persons with age 50 years or more (v) number of persons having higher education. Furthermore, this study also validates the effectiveness of local parameter estimation by comparing the global OLS method with the local GDR method. To the best of our knowledge, this is the first study that explores geographically varying relationships of COVID-19 deaths with various potential driving factors in India.

Rigorous analyses are performed to demonstrate the shortcomings of global technique (OLS) as compared to the local technique (GWR) in terms of several performance metrics. The OLS model only explains 71.9% of the variance of district-level COVID-19 deaths. It is found that the predictive efficiency and model accuracy are further enhanced by implementing the GWR method. The GWR model explains 97% of the variance of district-level COVID-19 deaths. Moreover, Moran’s I index verifies that no significant spatial autocorrelation is present in the residuals of the GWR model. Note that, a key advantage of such a local method is its capability to visualize the geographically varying heterogeneous relationships between the dependent and the independent variables. In other words, it enables us for a better understanding of relationships based on geographical contexts and study area’s known features.

The findings of this study reveal that there are strong positive relationships of COVID-19 deaths with the explanatory variables *PM*2.5 and $Tot\_population$ across the regions of the COVID-19 death hotspots in the western part of India. The positive association of COVID-19 deaths with long term exposure of $PM_{2.5}$ is consistent with the previous works^20,21^. Note that, long-term $PM_{2.5}$ exposure is substantially associated with some of the comorbidities (e.g. chronic lung disease, cardiovascular disease, etc.) that may lead to COVID-19 deaths^22,23^. Similarly, a positive association between COVID-19-related deaths and $Tot\_population$ is also observed in other studies^6,24^. However, the reverse association is found for these two variables ($PM_{2.5}$ and $Tot\_population$) in the other parts of India. The explanatory variable $HH\_With\_TCMC$ is found to be an important factor that may be a measure of the number of households with the upper class and rich people. A strong positive relationship is observed between $HH\_With\_TCMC$ and COVID-19 death in the hotspots of eastern and western parts of India (Kolkata, North 24 Parganas, Pune, Thane, Surat, Nagpur, etc.). Note that, in those hotspots, the value of $HH\_With\_TCMC$ is substantially high. An interesting observation reveals that a strong negative relationship exists between COVID-19 death and $Higher\_Edu$ in the eastern, central, and southern parts of India. It is expected that the higher educated people are well aware of the symptoms and the complications of COVID-19 that may lead to the fewer number of fatalities in those regions. Now, in some regions of the south-eastern part of India, the number of COVID-19 deaths is also seen to be high.

In those regions, significant positive relationships are found between $COVID-19$ deaths and $Tot\_population$, whereas significant negative relationships are observed for the variable $Higher\_Edu$.

This research work inherits certain shortcomings that need to be resolved in future research. For instance, there may have high possibilities of under-reporting in COVID-19 death counts that may introduce bias in the study^25^. Moreover, due to data unavailability, we were not able to include some significant district-level driving factors in our study, such as health care system quality, number of hospital beds, household income, and poverty data. Despite the above-mentioned shortcomings, this is the first study that explores geographically varying relationships of COVID-19 mortality with different socioeconomic, demographic, and environmental pollution related factors in India. This research work also highlights the significance of the geographically weighted regression in the geographical analysis of the health outcome of COVID-19 disease.

## Conclusion

COVID-19 pandemic is one of the most serious global public health catastrophe of the century. In this work, the geographically varying relationships between COVID-19 deaths and different potential driving factors are assessed across India. The geographical distribution of reported COVID-19 death cases is found to be heterogeneous over India. This heterogeneity in distribution is related to many underlying factors, including demographic, socioeconomic, and environmental pollution related variations between different parts of India. The GWR model makes it possible for the regression coefficients to differ across the geospace, creating geographical patterns about the strength of the relationship. The geographical heterogeneity and non-stationary of the relationships between COVID-19 deaths and the driving factors are demonstrated by mapping the local parameter estimates. The local parameter estimates reflect the quality of local model fitting and the nature of the association. The local method (GWR) yields better performance with smaller AICc as compared to the global method (OLS).

It should be noted that the impacts of other influencing factors (e.g. Meteorological factors) are not included in this work. This might be the direction for future studies. Moreover, in this study, currently we do not consider time evolution of variables, it is because for the dependent and the independent variables we may require more time series data for the effective temporal modelling. However, we plan to consider the time evolution of the variables for the future studies when more time series data will be available.
